# Leadless pacemaker implantation in a patient with a history of tricuspid edge-to-edge repair

**DOI:** 10.1016/j.hrcr.2025.07.012

**Published:** 2025-07-23

**Authors:** Zeynep Demirtakan, Julius Nikorowitsch, Anna Traub, Charlotte Eitel, Roland Tilz

**Affiliations:** 1 Department of Rhythmology, University Heart Center Lübeck, University Hospital Schleswig-Holstein, Lübeck, Germany; 2 Deutsches Zentrum für Herz-Kreislauf-Forschung (DZHK), Partner Site Hamburg/Kiel/Lübeck, Lübeck, Germany

**Keywords:** Leadless pacemaker, Tricuspid edge-to-edge repair, Valve-sparing pacing, Tricuspid disease, Pacemaker implantation

**Key Teaching Points**

• Traditional transvenous pacemaker leads can worsen tricuspid regurgitation by interfering with valve function, particularly in patients with prior tricuspid valve interventions. Leadless pacemakers provide a valuable valve-sparing alternative, reducing the risk of leaflet impingement, lead-related complications, and device-induced endocarditis.
• Pacemaker implantation in patients with prior transcatheter edge-to-edge tricuspid valve repair requires careful procedural planning. Navigating a leadless pacemaker past implanted clips can be technically demanding, necessitating real-time echocardiographic and fluoroscopic guidance to ensure optimal device placement and avoid damaging the repair structure.
• Fluoroscopic imaging alone may be insufficient for optimal pacemaker placement in patients with prior valve repair. In this case, transthoracic echocardiography provided critical guidance for safe navigation and implantation, confirming adequate device positioning while maintaining a safe distance from the existing tricuspid valve clips.

## Introduction

There is a complex interplay between pacemaker implantation and tricuspid valve disease. Although pacemakers can contribute to worsening tricuspid regurgitation through various mechanisms, the presence of a repaired or replaced tricuspid valve can pose challenges for lead implantation. In patients with tricuspid valve repairs or prosthetic valves, endocardial leads should be avoided in favor of valve-sparing techniques.^1^^,^^2^ Leadless pacemakers have increasingly replaced transvenous pacemakers in select populations, particularly in patients with significant tricuspid valve disease or prior valve interventions.

We present the case of a patient who underwent transcatheter edge-to-edge repair (TEER) and subsequently developed an indication for pacemaker implantation. The patient was successfully treated with a leadless pacemaker, implanted under both echocardiographic and angiographic guidance. The procedure resulted in optimal pacemaker function and an adequate distance between the implanted clips and the device, ensuring satisfactory performance.

## Case report

An 83-year-old female patient was referred to our clinic with symptomatic second-degree atrioventricular (AV) block type 2. In the past few months, she had experienced recurrent presyncope, lightheadedness, and exertional dyspnea. During a routine visit to her physician, an electrocardiogram result confirmed second-degree AV block type 2, leading to the decision for pacemaker implantation.

Her medical history included TEER with the TriClip valve repair system 3 years ago because of recurrent cardiac decompensations caused by severe tricuspid regurgitation. Follow-up evaluations after the procedure revealed a good overall outcome, with minimal residual tricuspid insufficiency. One year ago, coronary angiography results revealed no significant stenosis, although right heart catheterization indicated mild pre- and post-capillary pulmonary hypertension. In addition, she had a history of a transient ischemic attack in 2012, recurrent deep vein thromboses, and pulmonary embolism. Her chronic conditions included diabetes mellitus, arterial hypertension, stage 2 chronic kidney disease, and heart failure with preserved ejection fraction. Echocardiography results revealed a left ventricle with preserved ejection fraction and normal diameter, including good results after tricuspid valve repair with only minimal residual tricuspid insufficiency. To prevent damage to the tricuspid valve, a leadless pacemaker was preferred over a conventional transvenous system. Even though there was no sinus node dysfunction and a VDD single-chamber leadless pacemaker could have been appropriate, the potential future need for atrial pacing led to a different decision. The AVEIR system was chosen as it is currently the only leadless pacemaker that allows for future upgrade from VVI to DDD mode through the addition of an atrial leadless pacemaker.

**Figure 1:**
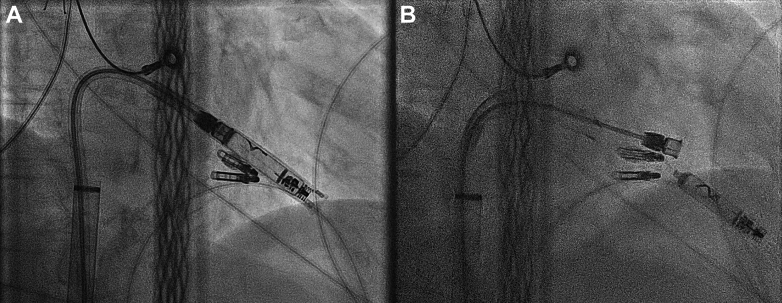
Fluoroscopic images in RAO 5° projection 186 revealing (**A**) positioning and (**B**) releasing of the leadless pacemaker. RAO = right anterior oblique.

**Figure 2:**
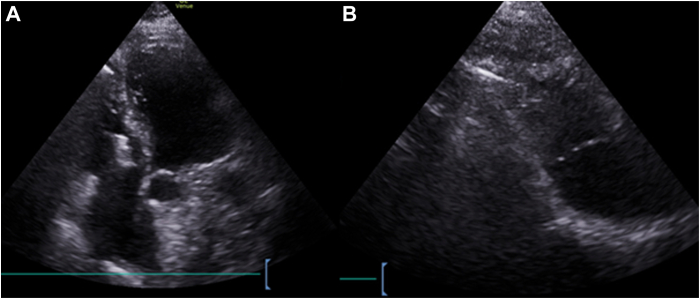
(**A**) Apical 4-chamber and (**B**) modified parasternal echocardiographic views revealing the positions of the tricuspid valve clips and the pacemaker device.

Before implantation, the patient’s oral anticoagulation therapy was paused for 24 hours. The procedure began with ultrasound-guided venous puncture of the right femoral vein. An Abbott introducer sheath (Abbott, 27 Fr) was inserted, followed by a delivery catheter preloaded with a leadless pacemaker, the AVEIR VR (Abbott). Passage of the tricuspid valve with the leadless pacemaker was challenging and only possible superior to the 2 previously implanted tricuspid valve clips. Therefore, the procedure was performed under both fluoroscopic and echocardiographic guidance (Figures 1 and 2). Right ventricular mapping was conducted to identify an optimal implantation site.

Owing to slight anatomical distortion, the best right anterior oblique (RAO) view was achieved at RAO 5°. The leadless pacemaker was positioned in a septal location and implanted by rotating the delivery handle, with a tactile click felt every 45° (Supplemental Video 1). Electrogram and impedance measurements were recorded at 1 and 1.5 turns. After 1.5 turns, the device entered the tether mode. A deflection stress test was performed to confirm secure fixation, followed by comprehensive electrical measurements to verify optimal device function. Once satisfactory measurements were obtained, the leadless pacemaker was successfully released (Supplemental Video 2).

The total procedure duration was 30 minutes. Postprocedural transthoracic echocardiography confirmed a safe distance of 20 mm between the clips and the pacemaker in the 4-chamber view, with no worsening of tricuspid regurgitation. The femoral puncture site was closed using a Perclose ProGlide (Abbott) vascular closure device and a figure-of-eight skin suture, allowing the patient to be mobilized within 4 hours. Pacemaker interrogation on the following day confirmed proper device function and stable measurements, including an electrode impedance of 840 Ω, an R-wave amplitude of 18 mV, and a threshold of 0.5 V at 0.4 ms. The patient remained stable postprocedure, with no complications.

## Discussion

Transvenous leads can worsen tricuspid regurgitation through various mechanisms, including leaflet impingement, perforation or adhesion, device-induced endocarditis, and electromechanical dyssynchrony. Since their introduction in 2016, leadless pacemakers have emerged as a safe alternative to transvenous systems. Some studies suggest that leadless pacemakers are associated with less deterioration of tricuspid regurgitation compared with endocardial leads.^1^^,^^3^

The implantation of intracardiac devices in patients with prior tricuspid valve interventions presents technical challenges, although case series have demonstrated its safety.^4^, ^5^, ^6^, ^7^ In patients with a history of TEER, lead implantation poses a risk of damaging the TEER device, and friction between the lead and the repair system may compromise lead integrity. Valve-sparing alternatives, such as epicardial leads, coronary sinus (CS) leads, His-bundle pacing (atrial side), and leadless pacemakers, have been explored, but there are limited data to establish the superiority of any particular approach. Although leadless pacemakers avoid direct mechanical interaction with the valvular apparatus, they may still contribute to tricuspid insufficiency due to ventricular dyssynchrony.^8^

When pacemaker implantation is required after tricuspid valve replacement, the European Society of Cardiology recommends considering valve-sparing pacing techniques.^1^ Valve-sparing pacing techniques in general include, for example, leadless pacing, surgical placement of epicardial leads, His-bundle pacing from the atrial aspect of the tricuspid annulus, and left univentricular pacing through the CS. In patients who have undergone TEER, transesophageal echocardiography-guided transvalvular lead placement, His-bundle pacing, or CS leads are suggested.^1^ After annuloplasty, either transvalvular leads or valve-sparing pacing alternatives are advised.^2^ Although the difference in device length (Micra AV: 25.9 mm vs Aveir: 38 mm) may have implications for tricuspid valve interference, the potential for future upgrade to dual-chamber pacing with the AVEIR system—should atrial dysfunction develop—was a key factor influencing the clinical decision, particularly in light of the patient’s advanced age and underlying conduction system disease.

In our patient, the procedure was performed under echocardiographic and angiographic guidance, successfully positioning the device after multiple attempts to navigate between the clips into the right ventricle. Postprocedural echocardiographic imaging demonstrated satisfactory results; however, standardized measurements and criteria for success remain undefined. Further studies comparing different approaches are necessary to improve procedural planning, guidance, and outcome assessment.

## Conclusion

In our case, we demonstrated that a leadless pacemaker can be safely implanted following a TriClip intervention. Although previous reports have described the use of the Micra leadless pacing system in similar scenarios, to our knowledge, this is the first report of successful implantation using the Aveir leadless pacemaker after TEER.

## Declaration of Generative AI and AI-Assisted Technologies in the Writing Process

During the preparation of this work, the authors used CHATGPT to improve language and readability, with caution. After using this tool/service, the authors reviewed and edited the content as needed and take full responsibility for the content of the publication.
