# Supplementary material for: Leadless pacemaker implantation in a patient with a history of tricuspid edge-to-edge repair
Source: HeartRhythm Case Rep. 2025 Jul 23;11(10):1026–9. doi: 10.1016/j.hrcr.2025.07.012 (PMC12666927; doi:10.1016/j.hrcr.2025.07.012)
Supplement: Supplementary Legends [file mmc3.docx]

**Supplementary material**

**Video 1:** Video showing fluoroscopic navigation of the leadless pacemaker through the triclips

**Video 2:** Video showing the device in right ventricle
